# Pd films on soft substrates: a visual, high-contrast and low-cost optical hydrogen sensor

**DOI:** 10.1038/s41377-018-0114-x

**Published:** 2019-01-09

**Authors:** Xiaoyi She, Yang Shen, Jianfang Wang, Chongjun Jin

**Affiliations:** 1 State Key Laboratory of Optoelectronic Materials and Technologies, School of Materials Science and Engineering, Sun Yat-sen University, Guangzhou, 510275 China; 2 School of Electronics and Information Technology, Sun Yat-sen University, Guangzhou, 510275 China; 3 Department of Physics, the Chinese University of Hong Kong, Shatin, Hong Kong SAR China

**Keywords:** Optical sensors, Imaging and sensing

## Abstract

For the rapid development of the hydrogen economy, a reliable and low-cost hydrogen sensor appears to be extremely important. Here, we first show that a palladium film deposited on polydimethylsiloxane (PDMS) can obtain an exceedingly high-reflectance contrast of 25.78 over the entire visible band upon exposure to 4 vol% hydrogen gas (H_2_) mixed with nitrogen gas. This high-reflectance contrast results from the surface deformation induced by the volume inflation after exposure to H_2_, leading to the transition of the near-specular surface to a diffusing surface. In addition, a change in brightness is readable by naked eye upon exposure to H_2_ with various concentrations from 0.6 to 1 vol% under the illumination of a fluorescent tube. Furthermore, this sensor possesses an excellent recyclability and quick response time of a few seconds. Compared with Pd nanostructure-based hydrogen sensors, this visual, high-contrast and low-cost sensor is of great potential for practical hydrogen sensing.

## Detecting hydrogen changes with the naked eye

A simple, low-cost sensor, designed for remotely detecting dangerous hydrogen levels, is extremely desired in hydrogen-related applications. Chongjun Jin at China’s Sun Yet-sen University, Jianfang Wang at the Chinese University of Hong Kong and co-workers have built a new type of optical hydrogen sensor by sputtering palladium on an elastic slab. As hydrogen was loaded, the palladium film would wrinkle deeply, namely, switching from the mirror-like surface to a coarse one. This phenomenon was not found in the palladium film on inelastic substrate. This hydrogen-induced surface deformation endows the sensor with a dramatically high reflectance change throughout the visible band and thus enables a distinct eye-readable change in color from dark to bright. These results offer great potential for developing inexpensive and sensitive eye-readable hydrogen sensors.

## Introduction

Hydrogen is expected to substitute traditional fossil fuels as a sustainable and clean energy carrier in future energy systems. However, the safety issue of this carrier is inevitable, since it easily explodes under volume concentrations ranging from 4% to 75% in air and a low ignition energy of 0.02 mJ^1^. Monitoring of hydrogen gas (H_2_) concentration is also essential to many industrial processes, such as in nuclear reactor safety, space applications, and coal mining^2,3^. Therefore, a cheap, simply operated, and a reliable hydrogen sensor is extremely important for hydrogen-related applications.

Palladium is widely used in hydrogen sensing owing to its reversible hydride formation and thermodynamic stability under ambient conditions. When absorbing/releasing hydrogen, Pd can form hydride phases in a reversible manner. This process is associated with a change in structural, electrical, and optical properties, providing the basis for designing Pd-based sensors. Conventional hydrogen detection is mainly based on electrical sensors, which have been demonstrated by Pd nanotubes^4^, micro/nanowires^5–8^, and thin films^9–12^ with break junctions. However, these sensors can generate electric sparks at the sensing point. Optical sensors can eliminate the potential risk of sparks because they can be read out remotely. In the presence of H_2_, the permittivity alteration of Pd leads to an optical contrast as a function of H_2_ concentration^13^. A variety of Pd-based hydrogen sensors based on the change in light intensity have been reported^14–19^. A single Pd film on a SiO_2_ substrate was earlier demonstrated for hydrogen sensing^14^. To improve sensitivity, Mg_2_Ni/Ti/Pd multilayer films were reported^18,19^. Meanwhile, plasmonic hydrogen sensors have been developed because the surface plasmon resonance (SPR) wavelengths and/or intensities are very sensitive to the dielectric changes caused by the hydrogenation of Pd in the vicinity of noble metal nanostructures^20–28^. Bimetallic Pd/Au metamaterials^20^ and Pd/Au multilayer perfect absorbers^21,22^ have been realized to improve sensitivity through the change in the relative intensity at the resonance. A large relative reflectance change of 880% at 4 vol% H_2_ was obtained on a perfect absorber within a short wavelength band^21^. In addition, many plasmonic sensors were based on spectral shifts, including Au/Pd heterodimers^23,24^, Pd nanoparticles/SiO_2_ film/Au nanodisks^25^, and (Au core)/(Pd shell) nanocrystals^26–28^. The sensing performances of those hydrogen sensors all depend on the properties of their materials and/or structures, which cannot be changed by adjusting measurement instruments. This dependence limits further improvement of the sensing performance. Meanwhile, the complicated structures require sophisticated fabrication processes or expensive fabrication equipment. Recently, visual (naked-eye detectable) sensing has attracted broad interest due to the easy operation demonstrated in bio^29^, ammonia^30^, and hydrogen sensors^31,32^. Prior works on visual hydrogen sensors were mainly based on permittivity variations of hydrogen-sensitive materials during hydrogenation. For example, Y/Pd multilayer films^31^ and Mg/Ti/Pd multilayer films^32^ were reported to form an eye-readable change in color due to the change in the dielectric functions of Y and Mg upon exposure to H_2_. Overall, all the Pd-based optical hydrogen sensors are on rigid substrates, mainly glass (silica) slides, and Pd film/structures are easily peeled off due to volume expansion after several hydrogenation/dehydrogenation cycles and form a buckle network^33^. How to avoid the self-peeling from the substrate for the Pd-based optical hydrogen sensor is a challenge. For that issue, a Pd film on an elastic substrate might be a good choice because it will release the surface stress caused by the volume expansion during the hydrogenation process^34^.

Elastic materials have been extensively applied in wearable devices^35–38^. In electric hydrogen sensors, elastic substrates were utilized to form cracks of the Pd film through stretching^11,12^. The Pd film on the elastic substrate might form surface deformations due to the volume expansion during the hydrogenation process, and these deformations are limited with rigid substrates. The surface deformations will enhance light scattering and reduce specular reflection, possibly enhancing reflectance contrast and eventually leading to wearable and visual optical hydrogen sensors.

Here, we report on a visual, high-contrast and low-cost optical hydrogen sensor comprising a thin Pd film on an elastic polydimethylsiloxane (PDMS) substrate, which is called a Pd-capped elastomer (PCE). The optical response of the PCEs to hydrogen gas is monitored by recording the reflection spectra of the sensors. It is very surprising that the change in reflectance occurs over more than 60.8% over the entire visible band upon exposure to 4 vol% H_2_ mixed with nitrogen gas (N_2_). The corresponding reflectance contrast exceeds 25.78 in the visible spectrum and is nearly three times as high as the best results for Pd-based hydrogen sensors ever reported^21^, and this result only occurs in a very narrow band. The PCE can also serve as a visual optical hydrogen sensor with conventional illumination equipment. These results cannot be explained by only the change in optical parameters, even with full hydride formation. Indeed, we find that the surface deformation induced by the volume inflation upon exposure to H_2_ leads to the transition of the near-specular surface to a diffusing surface. The diffusing surface dramatically reduces the specular reflection and therefore results in such a high-reflectance contrast. Based on light-scattering theory, the reflectance contrast of the sensor can be further enhanced by adjusting the collection angle of the detector and/or the distance between the incident point on the sensor and the detector.

## Results

### Sensing performance of the PCEs

**Fig. 1:**
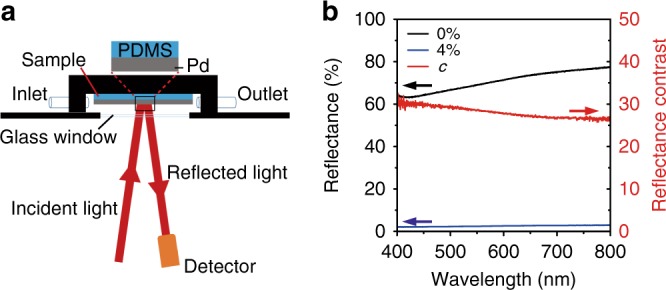
Hydrogen-induced reflectance change of a PCE. **a** Schematic of the optical measurement setup for the PCE mounted in a gas flow cell. **b** Reflectance spectra of the PCE with an 85-nm Pd film on exposure to 0 and 4% H_2_; the reflectance contrast spectrum defined as *c* = *R*_0%_/*R*_4%_ is also shown in this figure, where *R*_0%_ and *R*_4%_ represent the reflectances on exposure to 0 and 4% H_2_, respectively

The PCE samples comprised Pd films on PDMS substrates. Figure 1a presents a schematic of the reflection measurement setup for the PCEs on exposure to H_2_ mixed with N_2_ using a UV/Vis/NIR spectrometer (Lambda 950, PerkinElmer, USA). Figure 1b shows the reflection spectra of the PCE with an 85-nm Pd film under unpolarized excitation at an incidence angle of 8° after being exposed to 0 and 4% H_2_. Before exposure to H_2_, the reflectance exceeded 63.1% in the visible spectrum due to the metallic property of Pd^14^. However, after exposure to 4% H_2_, it is very surprising that the reflectance dropped dramatically to 2.95% throughout the entire visible band. Assuming that the Pd film is fully hydrogenated, the reflectance would still exceed 38.0% based on the FDTD simulation (Supplementary Fig. S1). This outcome indicates that this abnormal reduction in reflectance conflicts with the existing sensing mechanism based on the change in the optical parameter of the Pd film after hydrogenation. The absolute reflectance change *ΔR* = *R*_0%_–R_4%_ and reflectance contrast *c* = *R*_0%_/*R*_4%_ exceed 60.8% and 25.78%, respectively, over the entire visible band, where *R*_0%_ and *R*_4%_ represent the reflectance upon exposure to 0 and 4% H_2_, respectively. This outcome indicates that the sensor can achieve naked-eye detection and does not need any optical detector. Compared with any Pd film and Pd-related nanostructures on rigid substrates, this result is much better than in any existing reports (Supplementary Table 1).

**Fig. 2:**
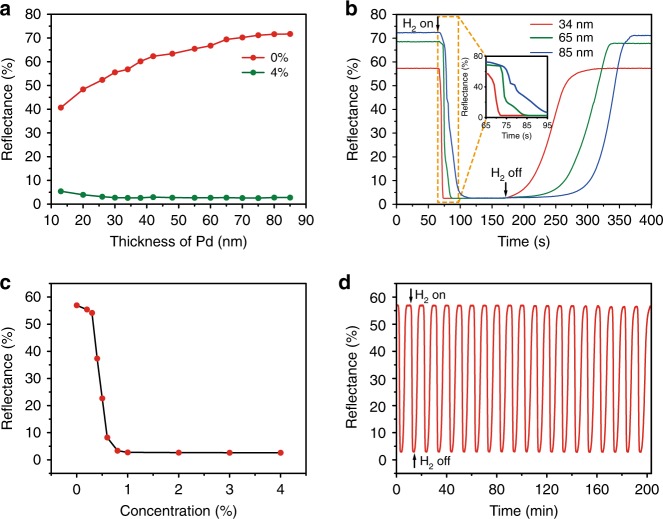
Performance of the PCEs as hydrogen sensors. **a** The relationship of the reflectance at 600-nm wavelength of the PCEs with various thicknesses of Pd films exposed to 0 and 4% H_2_. **b** Hydrogenation dynamics of the PCEs with various thicknesses (34, 65, and 85 nm) of Pd films exposed to 4% H_2_ at 600-nm wavelength. The onsets of H_2_ loading (66 s) and unloading (170 s) are indicated by the black arrows. For clarity, the inset shows in more detail the hydrogenation dynamics curves in the 65–95 s time range indicated by a dashed box. **c** Reflectance of the PCE at 600-nm wavelength on exposure to the hydrogen flows with concentrations varying from 0 to 4%. **d** Real-time optical responses of the PCE exposed to 0 and 1% H_2_ for 20 cycles at 600-nm wavelength. All of the total flow rates of the aforementioned experiments were 400 sccm. The thicknesses of the Pd films in **c** and **d** are 34 nm

Inspired by this result, we also studied the effect of the thickness of a Pd film on the reflectance upon exposure to H_2_. Figure 2a presents the relationships between the reflectance of the PCEs at 600-nm wavelength and the thicknesses of Pd films when exposed to 0% and 4% H_2_, respectively. The corresponding reflectance spectra in the visible band for the PCEs with various thicknesses of Pd films are shown in Supplementary Figure S2. The reflectance of the PCEs before hydrogenation becomes high at the same wavelength with an increase in Pd thickness from 13 to 80 nm; with the further increase in Pd film thickness, the reflectance is nearly the same. This result can be understood by the metallic property of Pd. If the Pd film is thicker than five times the skin depth (~13 nm), the reflectance of the film can be nearly thought to be the reflectance of bulk Pd. However, the reflection spectra of the PCEs exposed to 4% H_2_ are nearly the same when the Pd film is thicker than 30 nm. Therefore, the change in the reflectance at the same wavelength Δ*R* increases for the thicker Pd films and becomes nearly saturated at a thickness of 80 nm. In addition, the response times to hydrogenation/dehydrogenation of the PCEs with three various thicknesses are shown in Fig. 2b. The response time to hydrogenation is nearly 10 times faster than that of dehydrogenation. The increase in the Pd film thickness slows the response to hydrogen. The PCE with a 34-nm Pd film had the shortest response time of 7 s (inset of Fig. 2b) and was thus chosen to demonstrate the sensing performance as a hydrogen sensor. The reflectance spectra of the PCE under various H_2_ concentrations from 0% to 4% are shown in Supplementary Figure S3, indicating that the detection concentration is saturated in 1% H_2_ concentration. The relation of the reflectance at 600-nm wavelength to the concentration of H_2_ is illustrated in Fig. 2c. The linear detection concentration ranges from 0.3% to 0.6%. Furthermore, we also measured the reflectance of the PCE for incidence angles varying from 8° to 30° when exposed to 0 and 4% H_2_ (Supplementary Fig. S4). The changes in reflectance of the PCE during hydrogenation were nearly the same at various incidence angles, suggesting that our structure is angle-independent in this range of incident angle. The temporal response at 600-nm wavelength was also measured in twenty cycles, during which the hydrogen concentration was repeatedly switched from 0 to 1% (Fig. 2d). The reflectance change was nearly the same in each cycle. The reflectance spectra of the PCE for 20 cycles on exposure to 0 and 1% H_2_ were also measured (Supplementary Fig. S5). There was also no clear change in those twenty cycles throughout the visible band. Therefore, those optical responses of the PCE to H_2_ exhibited good recyclability under multiple measurements. To check the cross-sensitivity to other gases, we also measured the reflection spectra when a PCE was exposed to a mixture of air and H_2_ (Supplementary Fig. S6). As 4% H_2_ in air was loaded, the PCE exhibited the same reflectance contrast as that in N_2_ (Supplementary Fig. S6a), suggesting that our PCE detector can work well in the air environment (rich in oxygen). Compared with 4% H_2_ in N_2_, the response time of the PCE in 4% H_2_ mixed with air increased from 2 to 14 s, but the dehydrogenation time decreased from 172 to 10 s (Supplementary Fig. S6b). The temporal response at 600-nm wavelength was also measured in twenty cycles, in which the hydrogen concentration was repeatedly switched from 0 to 4% mixed with air (Fig. S6c). The reflectance in 4% H_2_ was nearly the same in each cycle, whereas the reflectance in 0% H_2_ slowly decreased from 58 to 56% after eight cycles and changed trivially in the remaining cycles. The difference between hydrogenation/dehydrogenation kinetics of H_2_ in N_2_ and H_2_ in air can be understood by competing surface reactions with oxygen. To further investigate the impact of the loading speeds and concentrations of H_2_ on the performance of this device, we measured the real-time reflectance of a PCE under varying flow rates and volume concentrations of H_2_ (Supplementary Figure S7a, b). It was found that the faster flow rate and higher concentration can both boost the hydrogenation process. Moreover, we tested the recyclability of the PCE under the sudden H_2_ loading (flow rate of 1000 standard cubic centimeters per minute (sccm)) with a high concentration (12% H_2_) for 20 cycles (Supplementary Figure S7c) to mimic a realistic hydrogen leakage. The result shows that even the repeated sudden stress can hardly deteriorate the optical responses of the PCE. Unexpectedly high contrast, excellent recyclability, and highly selective response and angle independence indicate that our PCE is very suitable for use as a visual optical hydrogen sensor.

### PCE as a visual hydrogen sensor

**Fig. 3:**
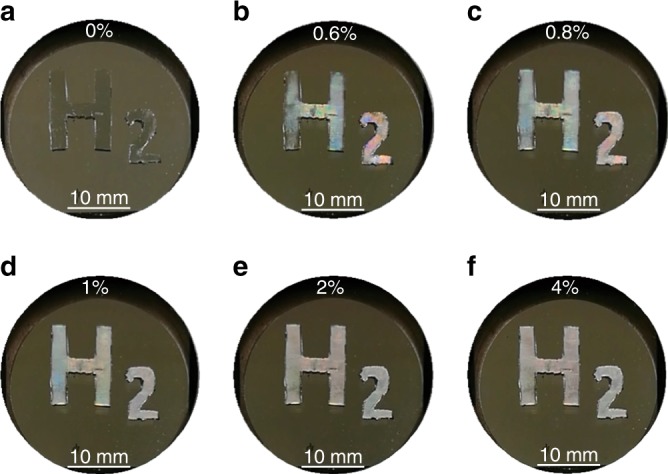
Naked-eye detectable hydrogen sensor. The photos of the “H_2_” pattern on exposure to H_2_ with various concentrations illuminated by fluorescent tubes. The “H_2_” pattern comprised a 34-nm Pd film on PDMS, while the other area was deposited with a 34-nm Pd film on quartz. The H_2_ concentrations are 0% in **a**, 0.6% in **b**, 0.8% in **c**, 1.0% in **d**, 2.0% in **e**, and 4.0% in **f**. The photos were taken using a mobile camera

The high-reflectance contrast of a PCE during hydrogenation and dehydrogenation processes indicates that it could serve as a visual hydrogen sensor. A carved “H_2_” pattern made from a PDMS slab was pasted on a circular quartz slide and then sputtered with a 34-nm Pd film. Figure 3 shows the color and brightness variations of that “H_2_” pattern exposed to H_2_ at various concentrations illuminated by fluorescent tubes. Before hydrogenation, the Pd film has a similar metallic appearance irrespective of the softness of the substrates, appearing gray (Fig. 3a). Upon hydrogen exposure, the “H_2_” pattern of the PCE became much brighter, unlike our expectation that the reduction in reflection would result in a dark color. It is clear that the “H_2_” pattern became brighter with the increase in the H_2_ concentration range from 0.6% to 0.8% (Fig. 3b, c). Meanwhile, abnormal shining colors on different regions reveal that the surface might have had a severe deformation, which could have diffracted the incident light. When the hydrogen concentration exceeded 1% (Fig. 3c–f), its further increase did not generate observable color and brightness variations, and the pattern looked whiter. This outcome can be understood by the saturation of hydrogen atoms in Pd. In the saturation state, the “H_2_” pattern became white, without color change. The brightening of the “H_2_” pattern looks similar to the phenomenon of a traffic sign illuminated by the headlight of an automobile, which can attract more attention than other visual sources without the surface scattering. These results point to a potential approach to the development of inexpensive and sensitive eye-readable hydrogen sensors.

## Discussion

### Surface deformation of the PCEs during hydrogenation

**Fig. 4:**
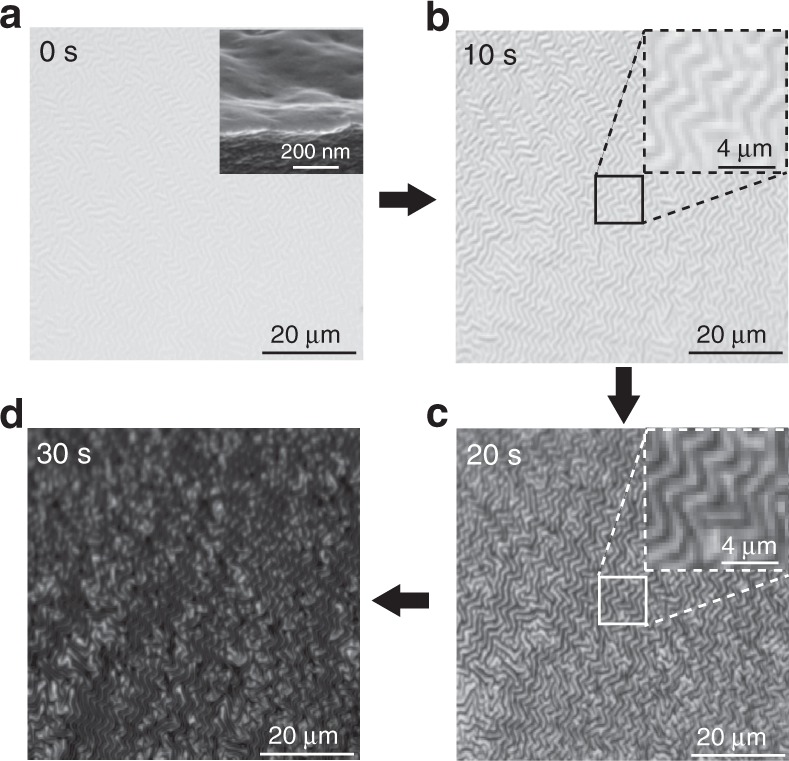
Surface deformation during the hydrogenation process. **a**–**d** Microscopic images showing the evolution of the surface deformation of the PCE with a 34-nm Pd film during the hydrogenation process. The insets are the tilted view of an SEM image at 0 s and the magnified images at 10 and 20 s, respectively. The concentration and flow rate of H_2_ were 4% and 400 sccm, respectively

To understand the abnormal reflectance and brightness changes of the PCEs, we inspected the temporal surface deformation of the PCE on exposure to H_2_ through an optical microscope, as shown in Fig. 4. Before hydrogenation (Fig. 4a), a number of slight surface corrugations appeared and can be seen clearly in the inset of the scanning electron microscopy (SEM) image. These surface corrugations result from the Ar plasma treatment in the Pd sputtering process^39^. When 4% H_2_ was introduced to the flow cell, both the surface smoothness and reflectance began to change (Fig. 4b). After 20 s, the surface corrugations became heavy, and the reflectance further decreased (Fig. 4c). Finally, the hydrogenation reached saturation at 30 s (Fig. 4d). The sample became dark and showed a high optical contrast between the hydrogenation and dehydrogenation states (Fig. 4a). More details of the hydrogenation/dehydrogenation process are recorded in Supplementary Video 1. For comparison, a Pd film with the same thickness sputtered on a quartz slide was studied (Supplementary Fig. S8). After exposure to 4% H_2_, the volume expansion of the Pd film cannot generate any observable surface distortion on the rigid substrate, and the sample showed only an approximate 11.0% decrease in reflectance. The result suggests that the elastic substrate plays a key role in the change of reflectance after the hydrogenation process. In detail, the amplification of the surface corrugations induced by Ar plasma bombardment during the hydrogenation process might be the reason for such a remarkable change in reflectance and brightening upon exposure to H_2_. Actually, for the flat PCE without any surface corrugations (namely, no Ar plasma treatment), it can also achieve the same reflectance contrast as that of the slightly corrugated PCE by Ar plasma treatment, but a longer response time as well as several “training cycles” are required in this case (details of the influence of the Ar plasma treatment on the sensing performance of the PCE can be seen in Supplementary Figure S9).

**Fig. 5:**
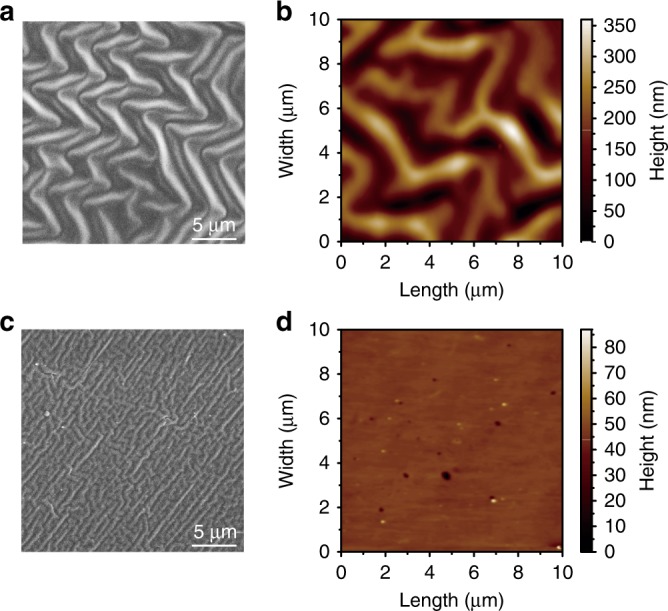
Inverse surface morphology of a Pd film on different substrates after hydrogenation. **a** SEM and **b** AFM images of the duplicated surface of the PCE with a 34-nm Pd film after exposure to 4% H_2_; **c** SEM and **d** AFM images of the duplicated surface of the 34-nm Pd film on quartz after exposure to 4% H_2_

The optical microscope image cannot disclose detailed information on the surface deformation of a PCE, while atomic force microscope (AFM) and SEM images of the surface should be better. However, it was difficult to observe the hydrogenated state of a PCE by SEM or AFM because the PCE recovered to its original position in a short period after the removal of H_2_. To observe the details of the surface deformation of the PCE after hydrogenation, we used hard PDMS to copy the morphology of a hydrogenated PCE. The schematic of the copy device is shown in Supplementary Figure S10. After the saturation of the hydrogenation of the PCE, we dropped hard PDMS on the PCE and kept H_2_ on until the hard PDMS was solidified. Figure 5a shows the SEM image of the duplicated PCE with a 34-nm Pd film in the fully hydrogenated state, which is the inverse morphology of the PCE after hydrogenation. The wrinkles on the surface of the duplicated PCE are clearly observable. Some random herringbone-type wrinkles appear. We also measured the wrinkle depth by AFM (Fig. 5b). The depths of the wrinkles are ~360 nm and agree well with the simulated results (380 nm, Supplementary Figure S11) by the finite element method (FEM) on the surface deformation of a PCE after hydrogenation (the details on the mechanical simulations can be found in the Materials and methods). Duplication of a 34-nm Pd film on quartz in the fully hydrogenated state was also observed by SEM and AFM (Fig. 5c, d), showing negligible surface deformation compared with the PCE. The wrinkles on the surface of the hydrogenated PCE can cause severe scattering, therefore reducing the strength of the specular reflectance and causing the “H_2_” pattern in Fig. 3 to brighten.

### Light scattering on the PCE during hydrogenation

**Fig. 6:**
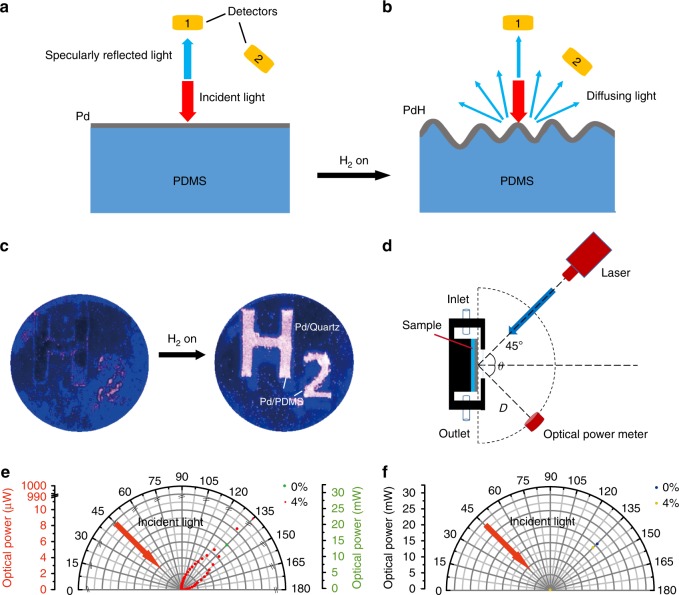
Sensing mechanism of the PCEs. **a** Schematic showing the capture of the specular reflection before H_2_ loading. Two detectors labeled 1 and 2 are placed in the directions of specular and diffuse reflections, respectively. In this case, the incident light is just specularly reflected by the flat Pd film (collected by detector 1), and there is no scattering. **b** Schematic showing the capture of the specular and diffusing reflections after H_2_ loading. In this case, the surface of the PdH_x_ film becomes a diffusing surface due to the volume dilation, where the specularly reflected light is dramatically suppressed (collected by detector 1) and the diffusing light appears (collected by detector 2). **c** Photos of the PCE illuminated by an expanded laser beam at a wavelength of 457 nm, where the “H_2_” pattern comprised a 34-nm Pd film on the PDMS, while the other area was deposited with 34-nm Pd films on quartz. **d** Schematic of the optical power measurements of the PCE illuminated by a laser beam (beam waist diameter of 0.73 mm) at a wavelength of 457 nm under 45° injection, where the size of the detection area was 5 × 5 mm^2^. The distance *D* between the optical power meter and the incident point on the sample is 20 cm. **e** Relationship between the measured optical powers of the reflected light of the PCE and the detection angles on exposure to 0% (green dot) and 4% H_2_ (red dots), respectively. **f** Relationship between the measured optical powers of the reflected light of the Pd film on a quartz substrate and the detection angles on exposure to 0% (blue dot) and 4% H_2_ (yellow dots)

In view of light-scattering theory, the mechanism of the abnormal reflectance change of the PCE can be explained using Fig. 6a, b. When the PCE is in the unloaded situation, only specular reflection occurs (collected by detector 1 in Fig. 6a), and there is no scattering. While in the loaded situation, the PCE becomes a diffusing surface due to the volume dilation, where the specularly reflected light dramatically drops (collected by detector 1 in Fig. 6b) and the diffusing light appears (collected by detector 2 in Fig. 6b). The transmittance of the PCE little changes at 4% H_2_ (Supplementary Figure S12), indicating that the incredible reduction in reflectance is mainly induced by the diffusion of the reflected light. For example, we fabricated an “H_2_” pattern comprising a 34-nm Pd film on the PDMS, while the other areas were deposited with a 34-nm Pd film on the quartz. An expanded laser beam was utilized to normally illuminate the sample. A schematic of the illumination system is shown in Supplementary Figure S13. A mobile camera (not in the direction of the specularly reflected light) was used to monitor the change in the diffusing reflection from the sample when exposed to 4% H_2_. As shown in Fig. 6c, during hydrogenation, the “H_2_” pattern brightened clearly, and the change in the brightness in other areas was inconspicuous. More details of the change in the scattering intensity can be seen in Supplementary Video 2. To further quantitatively explain the origins of the darkening phenomenon in Fig. 4 and brightening phenomenon in Figs. 3 and  6, respectively, we measured the angle-resolved optical powers of the reflected light from the PCE and the Pd film on a rigid substrate before and after hydrogenation, respectively. Figure 6d shows the schematic of the corresponding measurement system, where a PCE is illuminated by a laser beam at a wavelength of 457 nm under 45° injection, with a distance of 20 cm between the detector and the incident point on the PCE. In that configuration, the specular reflection and diffusing reflection components can be collected by placing the detector at the secularly reflected direction (135°) and diffusing directions (0–180^o^, except for 135°), respectively. Quantitatively, in the unloaded situation, as a laser beam with a power of 35 mW was injected, the powers of specular reflection and diffusing reflection components were 19 mW (green dot at 135°, Fig. 6e) and 0 mW (these dots are invisible due to the locations at the origin, Fig. 6e), respectively, suggesting that the PCE acts completely as a flat mirror due to its metal property. In the loaded situation, the power of the specular reflection of the corrugated PCE dramatically drops from 19 mW to 996 μW (red dot at 135°, Fig. 6e), while the diffusing reflection appears, and their power distributions are from 0.1 to 10.3 μW in the angle range of 45^o^–180° (red dots at 45^o^–180°, Fig. 6e). Therefore, one can observe either the darkening phenomenon based on the specular reflections or brightening phenomenon based on the diffuse reflections for the same sample. In addition, for a Pd film on the rigid substrate, the powers of the specular reflection were 21 mW (blue dot at 135^o^, Fig. 6f) and 18.3 mW (yellow dot at 135^o^, Fig. 6f) before and after hydrogenation, respectively, while there was no detectable diffusing reflection in those cases, suggesting that the Pd film on the rigid substrate is always in the mirror-like state regardless of whether in the loaded or unloaded situations. These phenomena confirm that the remarkable reduction in reflectance of the PCE after exposure to H_2_ comes from the diffusing reflection of the enhanced surface corrugations. This novel sensing mechanism completely differs from that for the existing optical hydrogen sensors comprising Pd or other hydrogen-sensitive materials on rigid substrates.

In summary, we have demonstrated a visual, high-contrast and low-cost optical hydrogen sensor comprising a Pd film on an elastic PDMS substrate. Greater than 60.8% change in the absolute reflectance from the PCE was observed over the entire visible band when exposed to 4% H_2_, and the corresponding reflectance contrast exceeded 25.78. Such an extremely high-reflectance contrast makes it easily detectable by the naked eye. The amplification of the surface corrugations induced by the volume inflation of the Pd films on the elastic substrates, namely, the transition of a specular surface to a diffusing surface during hydrogenation, plays a central role in the remarkable reflectance change. The reduction in the reflected light is turned to be the increase in the scattered light. In addition, the PCEs also show an excellent recyclability and quick response time. This novel sensing mechanism paves a new road to the design of visual optical gas sensors, especially hydrogen sensors. This high-performance sensor is simple and low cost, showing great potential in practical hydrogen-related applications.

## Materials and methods

### PCE fabrication

A PDMS slab was chosen as the elastic substrate because of its structural flexibility and chemical inertness^11^. To avoid the sticking of a silicon wafer to the cured PDMS, a monolayer of tridecafluoro-1,1,2,2-tetrahydrooctyl-1-trichlorosilane (TFOCS, Sigma-Aldrich) self-assembled on the entire surface of the cleaned silicon wafer in advance by placing the silicon wafer and a few drops of TFOCS in a Petri dish in a vacuum desiccator for 40 min. To prepare PDMS slabs, the base and the curing agent (Sylgard 184, Dow Corning, USA) were mixed in a Petri dish at a weight ratio of 10:1. The liquid mixture was then cast onto the silicon wafer with a monolayer of TFOCS. After bubbles were removed by placing the Petri dish in a desiccator, the sample was cured at 70 °C for 2 h in an incubator. An elastic PDMS slab was obtained by being peeled from the silicon wafer. Finally, a Pd film was deposited on the PDMS slab by magnetron sputtering to form a Pd-capped elastomer, namely, PCE. The base and deposition pressures under argon gas flow were 6 × 10^−4^ mbar and 0.01 mbar, respectively.

### Duplication of the surface morphology of the PCE after hydrogenation

To observe the surface deformation of PCEs on exposure to H_2_ via SEM, we have to duplicate the surface morphology via hard PDMS molding because the surface recovers to its original state in a short period after the removal of H_2_. First, a PCE was placed in a homemade flow cell with 4% H_2_ ventilation maintained for 3 h to increase its dehydrogenation time. To prepare hard PDMS, 3.4 g of (7–8% vinylmethylsiloxane)–(dimethylsiloxane) copolymer (VDT-731, Gelest, USA), 100 mg of 1,3,5,7-tetramethylcyclotetrasiloxane (SIT7900.0, Gelest, USA), and 50 mg of platinum divinyltetramethyldisiloxane (SIP6831.1, Gelest, USA) were mixed and degassed for 5 min. One gram of copolymer (HMS-301, Gelest, USA) (25–30% methylhydrosiloxane)–(dimethylsiloxane) was then added into the above mixture and quickly stirred within 1 min. Immediately, the PCE was placed in the copy device (Supplementary Figure S10) with hard PDMS in the dropper. After saturation of the hydrogenation of the PCE (within 1 min), the hard PDMS was dropped on the PCE, with H_2_ kept on until the hard PDMS was solidified (within 30 min). Finally, the hard PDMS was peeled from the surface of the PCE. A duplication of the surface morphology of the PCE in a fully hydrogenated state was obtained.

### Optical measurements

The optical responses to hydrogen gas were mainly monitored by recording the zero-order (specular) reflection spectra at room temperature. The schematic of the reflection measurement setup can be found in Fig. 1a. A variety of H_2_ (in N_2_) with different concentrations in volume ranging from 0 to 4% were generated by mixing the 4% H_2_ in N_2_ and pure N_2_ in a gas mixer. The mixture gas was introduced into a homemade flow cell and exhausted into a gas collector. Two mass flow controllers (CS100, Seven star, China) were utilized to precisely control the flow rates of H_2_ (in N_2_) and pure N_2_, respectively. A UV/Vis/NIR spectrophotometer (Lambda 950, Perkin Elmer, USA) was used to measure the reflection spectra. In the measurements, the unpolarized probing light illuminated the PCEs at an incidence angle of 8° through the transparent window at the top of the cell. The distance of the optical path between the detector and the sample was ~32 cm, and the area of the detector was 7 × 7 mm^2^. To eliminate the additional reflection from the glass window in the measurements, all reflection spectra were corrected by dividing them with the reflection spectrum of a silver mirror, which served as a reference only. The final reflection spectra of the PCEs were corrected by *R* = (*R*_sam_–*R*_emp_)/(*R*_mir_–*R*_emp_)×100%, where *R*_sam_, *R*_mir_, and *R*_emp_ represent the reflectances from the sample, the silver mirror, and the background when the cell was empty, respectively.

To further investigate the optical scattering properties of the PCEs after hydrogenation, a 457-nm laser beam was used to illuminate the sample at normal incidence, and the scattered light was collected by an optical power meter. Through moving the locations of the optical power meter along a semicircle with 20-cm radius and center located at the incident point of the laser on the PCE, the relation between the scattered light power and the scattered angle was determined. In that experiment, the detection area and the diameter of the laser beam waist were 5 × 5 mm and 0.73 mm, respectively.

The surface deformation of the PCE during the hydrogenation process was monitored by an optical microscope system. A 10X objective (MPlanFLX10, Olympus, Japan) with a numerical aperture (NA) of 0.4 was used to focus the white light from the tungsten–halogen lamp on the samples and collect the backscattered light. The observed images were finally recorded by a CMOS camera (Zyla-4.2P-USB3, Andor, UK).

### FDTD simulations

The finite-difference time-domain (FDTD) method (FDTD Solutions, Lumerical Solutions, Canada) was employed to simulate the reflection spectra of PCEs. Two plane waves with mutually perpendicular polarizations (natural light) were used to excite the PCEs with an area of 4 × 4 μm^2^. Periodic boundary conditions were applied on both the *x*- and *y*-axes. The mesh sizes of the metal region were set to 10 nm×1 nm×5 nm along the *x*-, *y*-, and *z*-axes, respectively. The permittivities of the Pd and PdH_x=1_ nanofilms in the visible region were taken from ref. ^40^. The refractive index of the PDMS substrate was 1.45^41^.

### FEM simulations

Finite element method (FEM) simulations were used to predict the surface deformation of the PCE in the presence of 4% H_2_. A unit cell was modeled as a 3D structure comprising two layers: a Pd film (thickness of 34 nm) and an underlying PDMS layer (thickness of 4 μm). The two herringbone-type ridges with 20-nm height were created to mimic the initial surface corrugations of the PDMS as well as the Pd film. The linear expansion coefficients in the *x*-, *y-*, and *z*-directions of the Pd film in 4% H_2_ were set to 1.04^42^. Periodic boundary conditions were utilized in the *x-* and *y-*directions. In addition, the fixed constraint was applied in the bottom of the PDMS. The Young’s modulus and Poisson ratio of PDMS were set as *E*_PDMS_ = 1143.7 KPa (measured by an electronic universal tester, WD-5A, China) and *σ*_PDMS_ = 0.49 (taken from ref. ^43^).

## Supplementary information

supplemental material

video 1

video 2
