# Supplementary material for: Pd films on soft substrates: a visual, high-contrast and low-cost optical hydrogen sensor
Source: Light Sci Appl. 2019 Jan 9;8:4. doi: 10.1038/s41377-018-0114-x (PMC6325063; doi:10.1038/s41377-018-0114-x)
Supplement: Supplementary file 1 — supplemental material [file 41377_2018_114_MOESM1_ESM.docx]

Supporting information

**Pd Films on soft Substrates: a Visual, high-contrast and Low-cost** **optical Hydrogen Sensor**

Xiaoyi She^1, 2,^*, Yang Shen^1,^*, Jianfang Wang^3^ *&* Chongjun Jin^1^

^1^State Key Laboratory of Optoelectronic Materials and Technologies, School of Materials Science and Engineering, Sun Yat-sen University, Guangzhou 510275, China. ^2^School of Electronics and Information Technology, Sun Yat-sen University, Guangzhou 510275, China. ^3^Department of Physics, The Chinese University of Hong Kong, Shatin, Hong Kong SAR, China. * These authors contributed equally to this work. Correspondence and requests for materials should be addressed to C.J.J. (Email: jinchjun@mail.sysu.edu.cn) and J.F.W.(Email: jfwang@phy.cuhk.edu.hk).

**
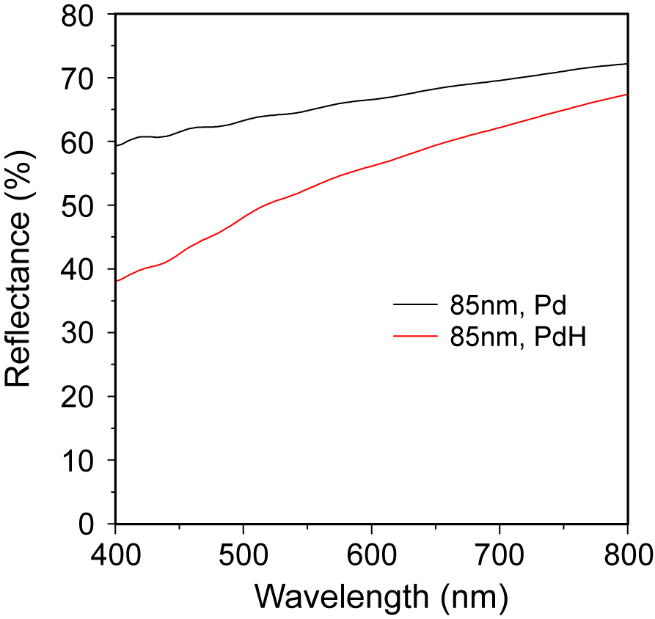
**

**Figure S1 Simulated reflection spectra of a PCE before and after hydrogenation without surface deformation based on the FDTD method.** The incidence angle is 8^o^. The thickness of the Pd film is 85 nm.

**
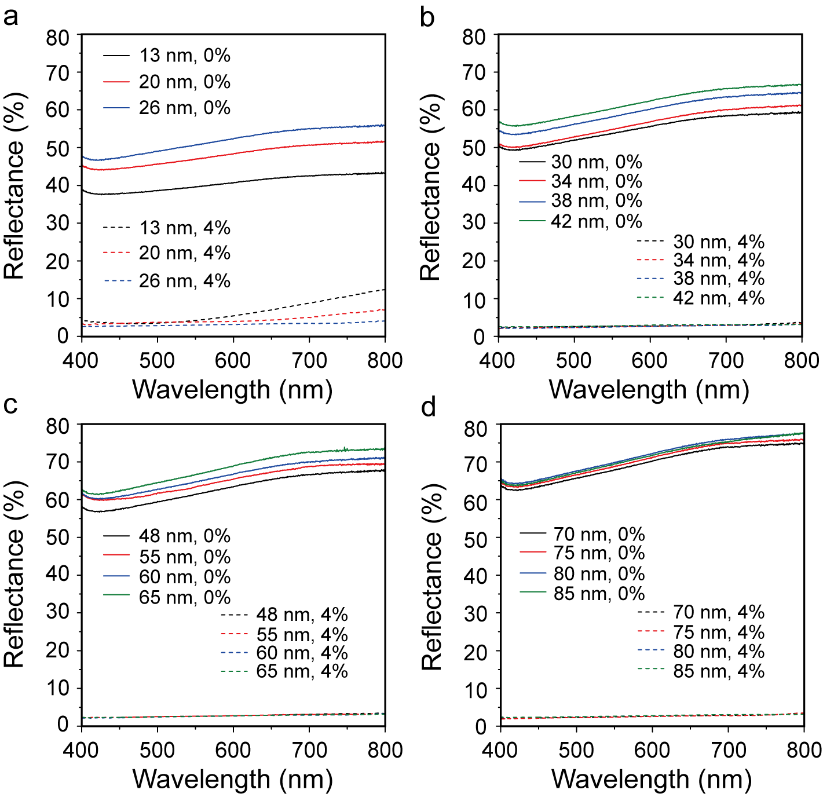
**

**Figure S2 Thickness-varied reflectance spectra of the PCEs.** The thicknesses of the Pd films are 13, 20 and 26 nm in Figure S2**a**, respectively; the thicknesses of the Pd films are 30, 34, 38 and 42 nm in Figure S2**b**, respectively; the thicknesses of the Pd films are 48, 55, 60 and 65 nm in Figure S2**c**, respectively; the thicknesses of the Pd films are 70, 75, 80 and 85 nm in Figure S2**d**, respectively.

**
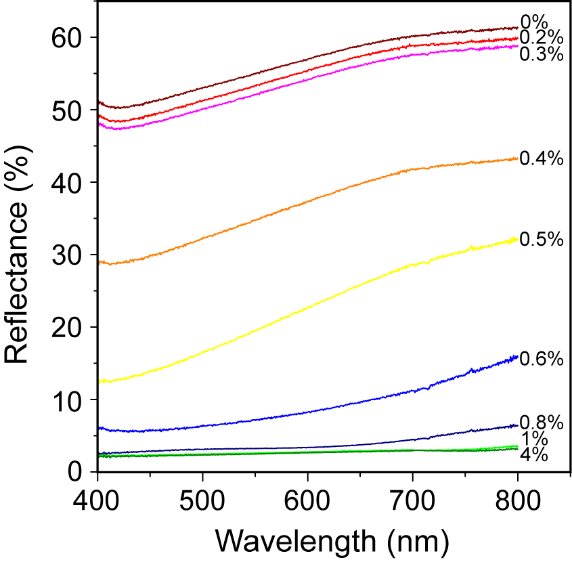
**

**Figure S3 Reflection spectra of the PCE exposed to different H_2_ concentrations.** The H_2_ concentration was varied from 0% to 4% at a total flow rate of 400 sccm. The thickness of the Pd film is 34 nm.

**
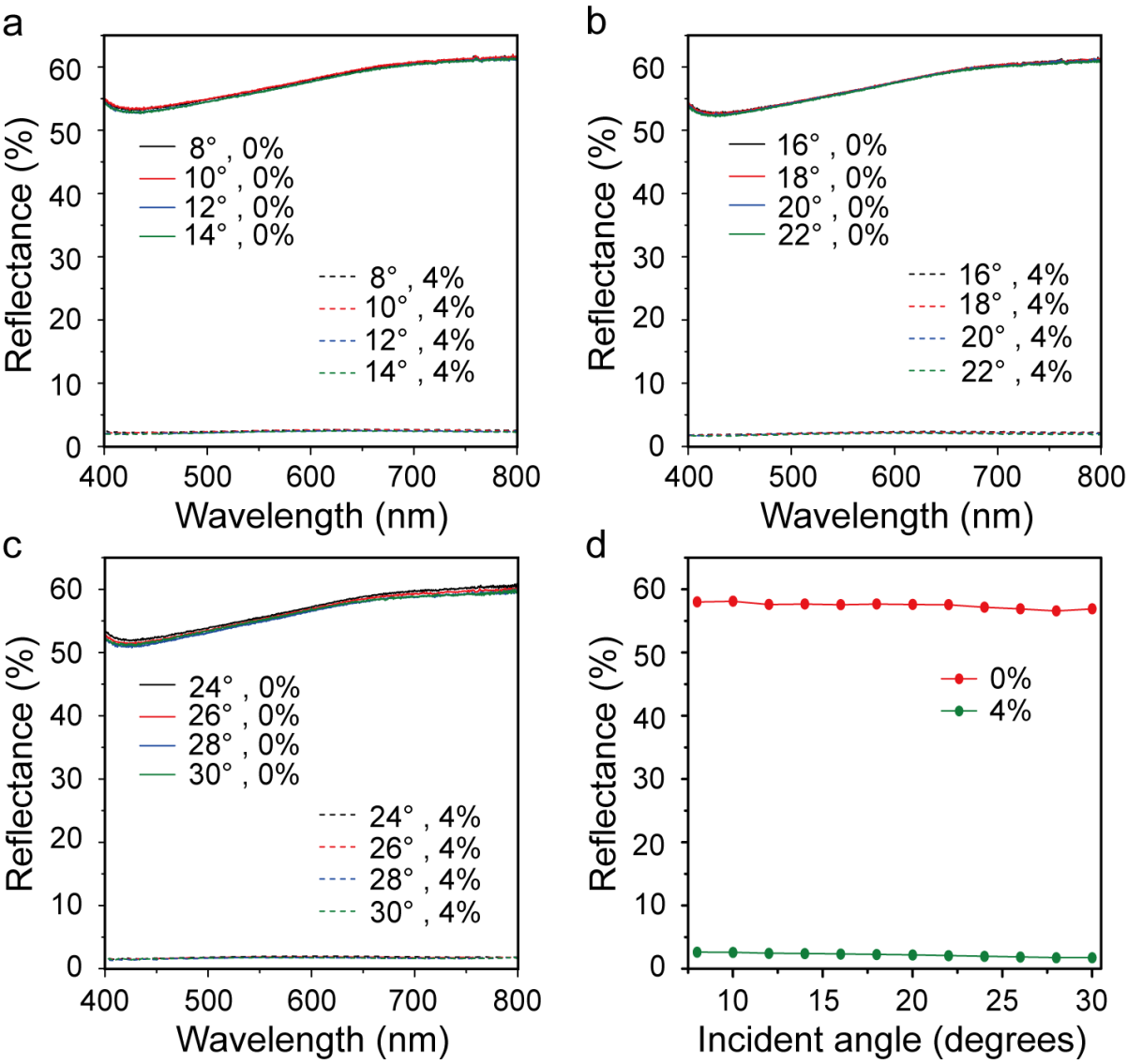
**

**Figure S4 Angle-resolved reflectance spectra of the PCEs.** (**a-c**) The incidence angles are 8°, 10°,12° and 14° in Figure S5a, 16°, 18°, 20° and 22°in Figure S4b, 24°, 26°, 28° and 30° in Figure S4c, respectively. (**d**) Reflectance of the PCEs as functions of the incidence angles at the wavelength of 600 nm for 0 vol% and 4 vol% H_2_.

**
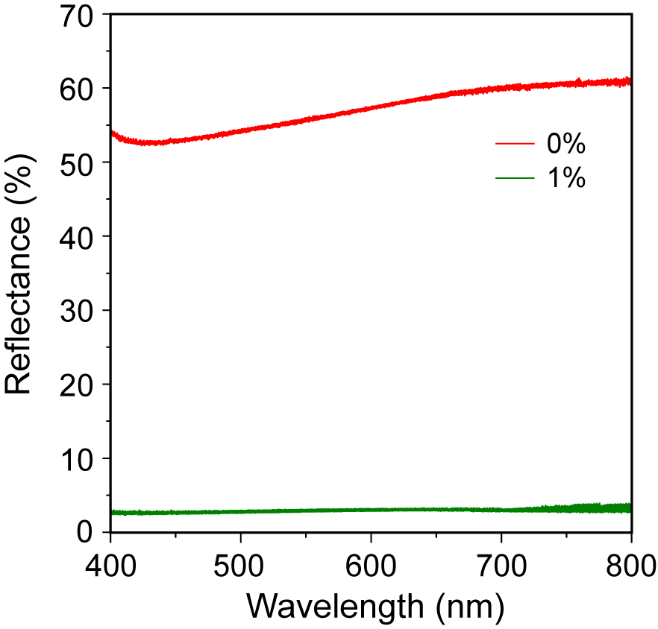
**

**Figure S****5 Recyclability test of a PCE**. The reflection spectra of the PCE with a 34-nm Pd film on exposure to 0% and 1% H_2_ alternatively for 20 cycles.

From Figure S5, the reflection spectra overlap with each other. There is no clear change within 20 cycles. These experimental results indicate that the response of the PCE to H_2_ exhibits excellent recyclability.


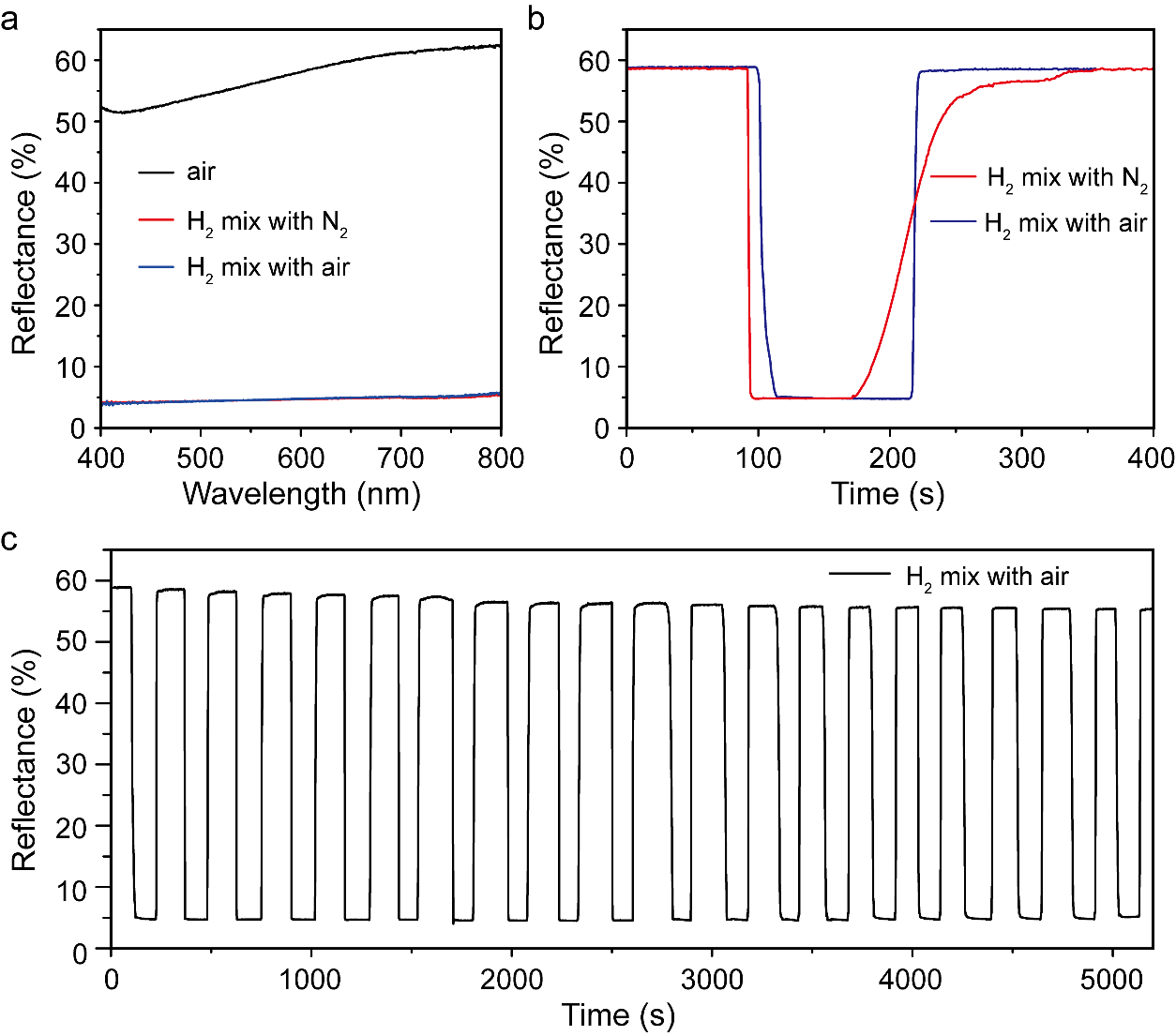


**Figure S6 Cross-sensitivity measurements.** (a) Reflection spectra of the PCE with a

34-nm Pd film on exposure to 4% H_2_ in N_2_ (red) and air (blue), respectively. (b) Temporal response of the PCE with a 34-nm Pd film on exposure to 4% H_2_ in N_2_ (red) and air (blue), respectively. (c) Real-time optical responses of the PCE exposed to 4% H_2_ in air and recovered by air for 20 cycles at the wavelength of 600 nm. All of the total flow rates of the aforementioned experiments are 1000 sccm. The 4% H_2_ in air is mixed with 8% H_2_ in N_2_ and air.


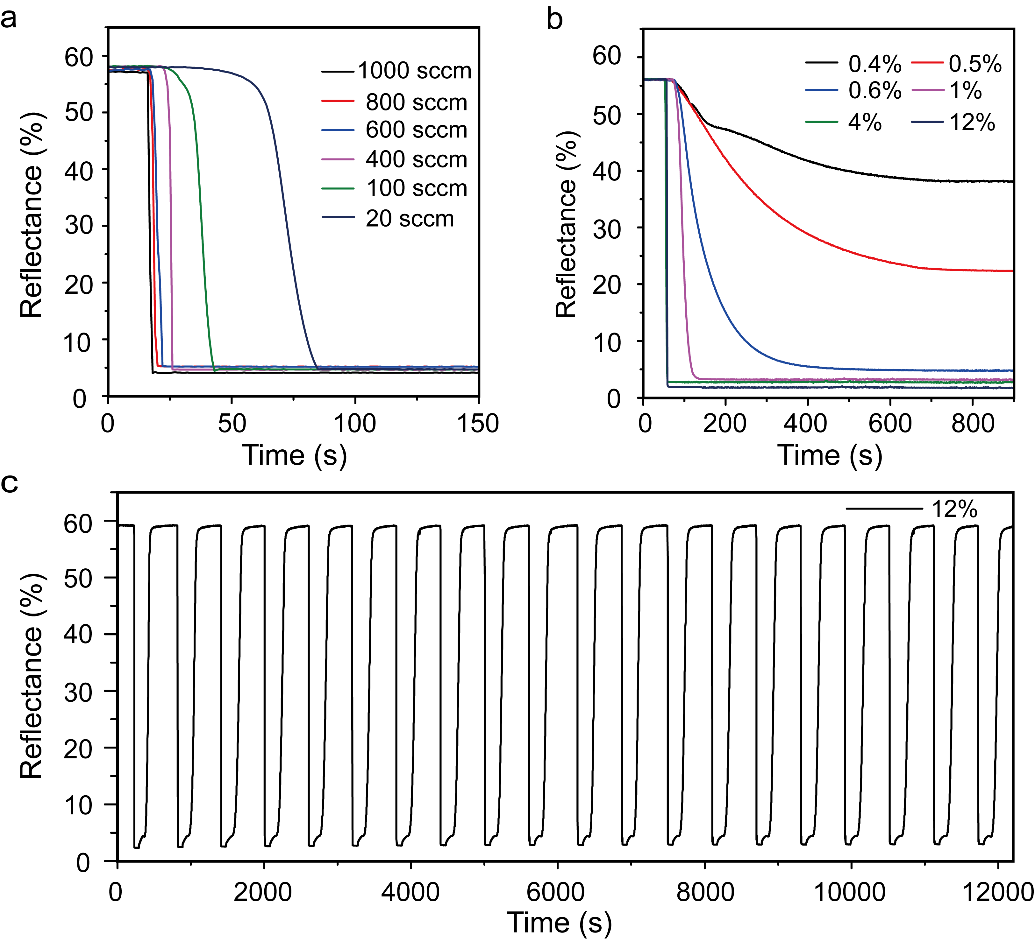


**Figure S7 Hydrogenation dynamics and** **recyclability of the PCE under sudden H_2_ loading with high concentration.** (a) Time-resolved optical responses at *λ* =600 nm of the PCE with a 34-nm Pd film when exposed to 4% H_2_ in N_2_ with varying flow rates. As the flow rate is increased from 20 to 1000 sccm, the response time of hydrogenation becomes faster from 56 to 2 s. (b) Time-resolved optical responses at *λ* =600 nm of the PCE with a 34-nm Pd film when exposed to 400-sccm H_2_ in N_2_ with varying concentrations. As the concentration is increased from 0.4% to 12%, the response time of hydrogenation becomes faster from 536 to 2 s. (c) Real-time optical responses at *λ* =600 nm of the PCE exposed to 0 and 12% H_2_ under the flow rate of 1000 sccm for 20 cycles.

**
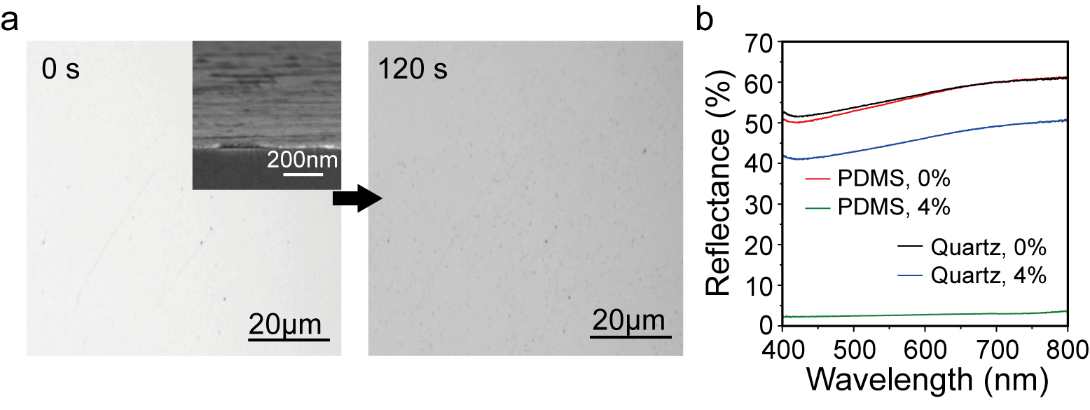
**

**Figure S8 Optical response of a 34-nm Pd film on quartz.** (**a**) Microscopic images showing the hydrogenation process of a 34-nm Pd film on a quartz slide observed by an optical microscope having a 10-fold objective lens. The inset is an SEM image of the sample. The concentration and flow rate of H_2_ are 4% and 400 sccm, respectively. (**b**) Reflection spectra of the 34-nm Pd film on the quartz slide and PDMS at 0% and 4% H_2_, respectively.

Figure S8a shows the hydrogenation process of a 34-nm Pd film on a quartz slide. Compared to a PCE, the Pd film is flat on the quartz before hydrogenation. When exposed to 4% H_2_, the sample became slightly dark without observable surface deformations after saturation. The corresponding reflection spectrum is shown in Figure S8b. In contrast to the PCE, the Pd film on the quartz displays only 11.0% reduction in reflectance, indicating that the permittivity alteration and volume expansion of the Pd film have limited effect on the reflectance. In addition, the reflection spectra of the 34-nm Pd film on the quartz almost coincides with that of the PCE at 0% H_2_. So the influence of the slight surface corrugations of the PCE on reflectance can be ignored before hydrogenation.


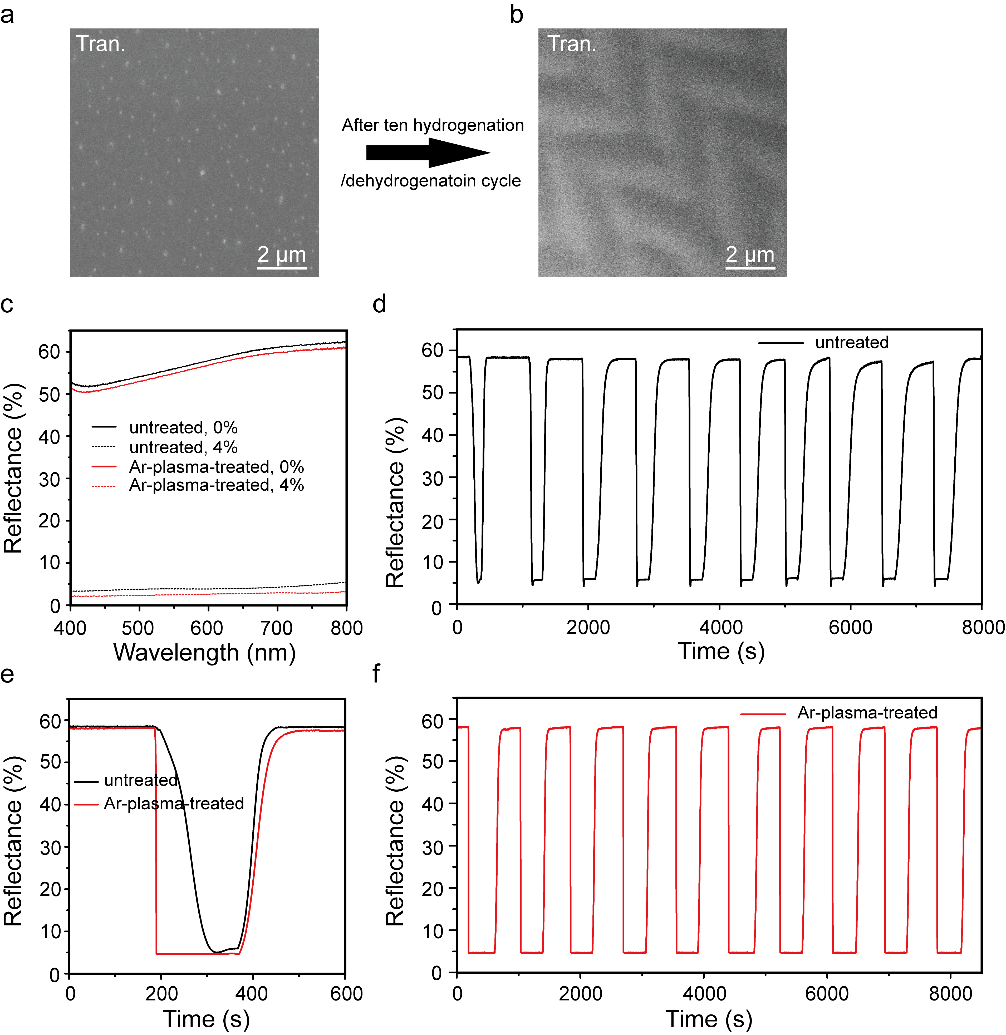


**Figure S9 Influence of the Ar plasma treatment on the sensing performance of the PCE.** (a) SEM image of a PCE without the Ar plasma treatment. The PCE was prepared by peeling off a flat Pd film deposited on a silicon wafer and transferring it onto a PDMS substrate. It exhibits no surface corrugations. (b) SEM image of the same PCE after 10 hydrogenation/dehydrogenation cycles, showing some slight surface corrugations. (c) Reflection spectra of Ar-plasma-treated PCE (red) and untreated PCE (black) with the same 34-nm Pd films when exposed to 0% and 4% H_2_ in N_2_, respectively. (d) Real-time optical responses at *λ* =600 nm of the untreated PCE exposed to 0 and 4% H_2_ for 10 cycles. (e) Comparison on the response times of Ar-plasma-treated and untreated PCEs in the first cycle, respectively. The response times are 7 and 138 s for Ar-plasma-treated and untreated PCEs, respectively. (f) Real-time optical responses at *λ* =600 nm of the Ar-plasma-treated PCE exposed to 0 and 4% H_2_ for 10 cycles. From the Fig. S9c, the untreated PCE has almost the same optical contrast with that of the Ar-plasma-treated one. But it requires a longer response time and several “training cycles” that introduces the slight surface corrugations as shown in Fig. S9b.

**
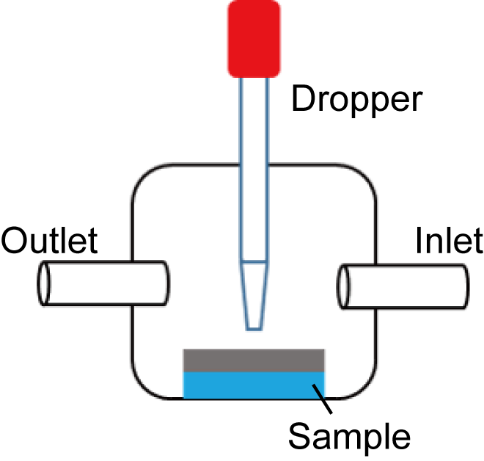
**

**Figure S10 Schematic of surface morphology duplication.** Hard PDMS was dropped on a PCE to duplicate its surface deformation after hydrogenation.

**
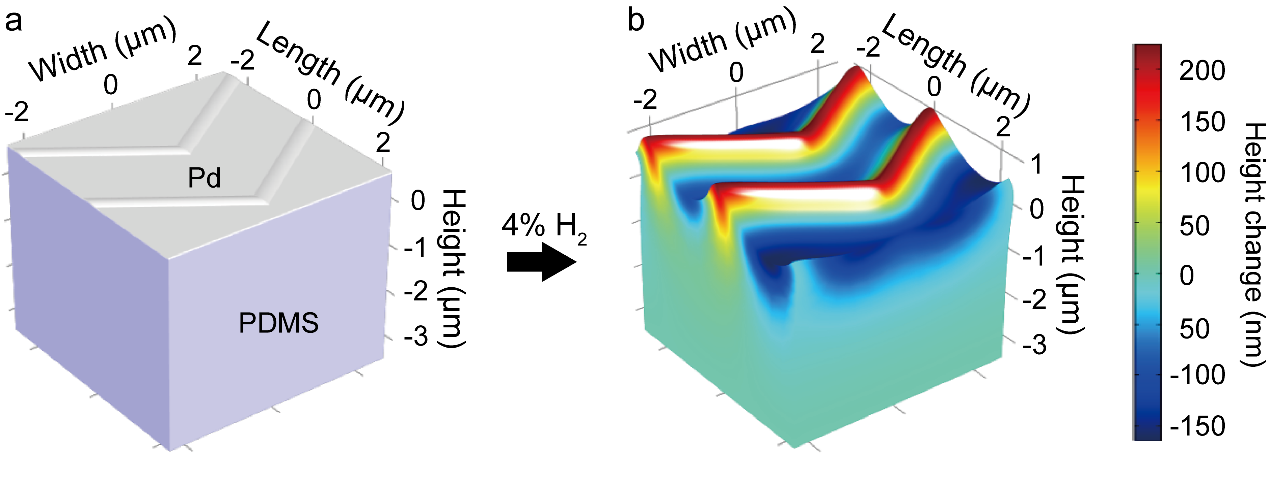
**

**Figure S11 Simulated surface deformation of a PCE when exposed to 4% H_2_ based on finite element method.** (a) Model of a PCE with a 34-nm Pd film before hydrogenation. Two herringbone-type ridges with the depth of 20 nm were created to mimic the initial surface corrugations of the PDMS and the Pd film, respectively. (b) Simulated surface deformation of the PCE when exposed to 4% H_2_. The wrinkles are amplified and their depths are around 380 nm at the equilibrium state.

**
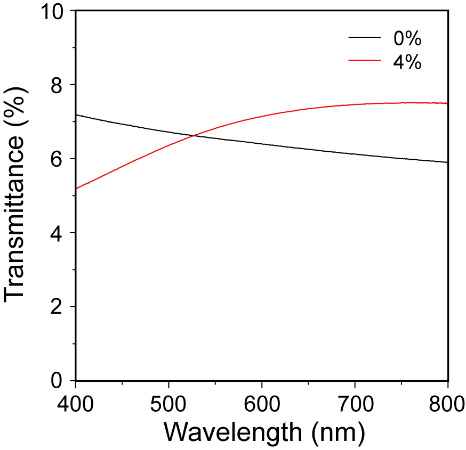
**

**Figure S12 Transmission spectra of the PCE with a 34-nm Pd film upon exposure to 0% and 4% H_2_, respectively.** It shows less than 3% change in transmittance.


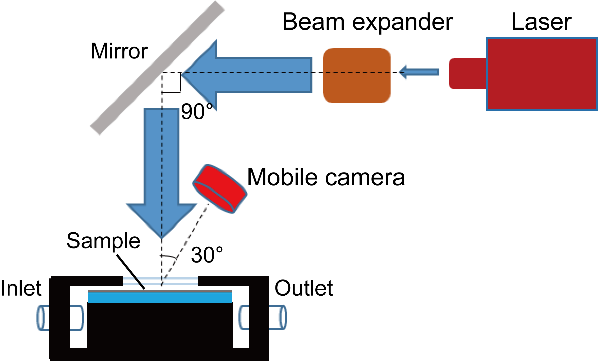


**Figure S13 Schematic of the setup where the sample with an “H_2_” pattern (Figure 6b).** It was illuminated by an expanded laser beam at the wavelength of 457 nm at normal incidence. A mobile camera mounted at the side of the flow cell was used to record the scattering light from the sample during hydrogenation.

**Table S1 Summary on the performance of the hydrogen sensors based on intensity change**

| Reference*^a^* | Nanostructures | Absolute intensity change Δ*I* | Optical contrast *I*_loaded_*/I*_unloaded_ or *I*_unloaded_*/I*_loaded_ | Response time for hydrogenation/s | Repeatability /cycle |
| --- | --- | --- | --- | --- | --- |
| Giessen, Appl. Phys. Lett. 2006, 89, 021913−021916 | Mg_2_Ni/Ti/Pd films | 48% at 653 nm -689 nm  @5%H_2_ | 786% at 653 nm -689 nm  @5%H_2_ | <75 | >20 |
| Giessen, Nano Lett. 2011, 11, 4366–4369 | Perfect absorber | 4.4%at 650 nm  @4%H_2_ | 980% at 650 nm  @4%H_2_ | 10 - 50 | >3 |
| Giessen, Nano Lett. 2014, 14, 1140−1147 | Y rod array | 23% at 1720 nm @5%H_2_ | 170% at 1720 nm  @5%H_2_ | 50 | – |
| Zayats, Adv. Mater. 2014, 26, 3532–3537 | Pd core/Au shell nanorod | – | 140% at 820 nm  @2%H_2_ | >240 | – |
| Giessen,Nano Lett. 2015, 15, 7949−7955 | Pd/Mg/Ti disk | – | 213% at 560 nm  @4%H_2_ | few minutes | poor  (oxidation) |
| Giessen, ACS Sens. 2016, 1, 1148−1154 | Perfect absorber | 4% at 950nm @4%H_2_ | 500% at 950nm  @4%H_2_ | – | >2 |
| our work | Pd-coated elastomer | >60.8% at 400 nm – 800 nm @4%H_2_ | 2578% at 400 nm – 800 nm  @4%H_2_ | 7 | >20 |

*^a^*Only the last name of the corresponding author or a representative corresponding author is given
